# Selecting HIV infection prevention interventions in the mature HIV epidemic in Malawi using the mode of transmission model

**DOI:** 10.1186/1472-6963-10-243

**Published:** 2010-08-19

**Authors:** Kenneth Maleta, Cameron Bowie

**Affiliations:** 1 Department of Public Health, Division of Community Health, College of Medicine, University of Malawi, Blantyre, Malawi

## Abstract

**Background:**

Malawi is reassessing its HIV prevention strategy in the light of a limited reduction in the epidemic. No community based incidence studies have been carried out in Malawi, so estimates of where new infections are occurring require the use of mathematical models and knowledge of the size and sexual behaviour of different groups. The results can help to choose where HIV prevention interventions are most needed.

**Methods:**

The UNAIDS Mode of Transmission model was populated with Malawi data and estimates of incident cases calculated for each exposure group. Scenarios of single and multiple interventions of varying success were used to identify those interventions most likely to reduce incident cases.

**Results:**

The groups accounting for most new infections were the low-risk heterosexual group - the discordant couples (37%) and those who had casual sex and their partners (a further 16% and 27% respectively) of new cases.

Circumcision, condoms with casual sex and bar girls and improved STI treatment had limited effect in reducing incident cases, while condom use with discordant couples, abstinence and a zero-grazing campaign had major effects. The combination of a successful strategy to eliminate multiple concurrent partners and a successful strategy to eliminate all infections between discordant couples would reduce incident cases by 99%.

**Conclusions:**

A revitalised HIV prevention strategy will need to include interventions which tackle the two modes of transmission now found to be so important in Malawi - **concurrency and discordancy**.

## Background

HIV infection prevention has been largely unsuccessful in many sub-Saharan countries including Malawi, where HIV prevalence remains high despite prevention activities [1,2]. In Malawi the current estimate for 2008 is 12% in 15-49 year olds [3]. Any reassessment of a prevention strategy as is happening in Malawi requires knowledge of where new HIV infections are arising [4]. Sources of new HIV infections can be identified in places where community based incidence studies have been carried out [5]. None such study has been carried out in Malawi. Estimating incidence in difference groups of the population can be done using mathematical models such as the "Mode of Transmission" model distributed by UNAIDS and recommended by their Reference Group on Estimates, Modeling and Projections [6,7].

Three things make it timely to review national HIV prevention strategies. Firstly, epidemiologists have belatedly come to realise the importance of concurrent sex partners in the spread of HIV infection. Until recently transmission models, while taking the importance of multiple partnerships into account, failed to include the magnitude of the spread within concurrent partnerships in the early stages of infection. An example of this failure is in a paper written in 1989 which wrongly anticipated a heterosexually spread epidemic in the UK because the difference between serial monogamous multiple partnerships and concurrent partnerships was not realised [8].

Secondly, the very high transmission risk in the early infection period was not fully realised until the paper from Rakai in 2005 [9]. The study showed the ten fold increase of risk of infection in early infection as compared to the base line rate which occurred from about five months after infection until the development of AIDS.

Thirdly, the belief that deep seated cultural practice of polygamy and concurrent sexual partnerships in Africa could not be changed was disproved [10]. The profound effect of a hard hitting (zero-grazing) campaign in Uganda has been compared to trends in Malawi. Given the similar (but different) cultures of Uganda and Malawi there is no reason why a hard hitting campaign would not work here [11].

The Mode of Transmission spreadsheet provides a simple means of assessing the impact of HIV prevention measures singly and in combination. This paper assesses the impact using a set of input variables to accurately reflect what is likely to be happening in Malawi. For this exercise absolute numbers are not as important as relative ones because the exercise is looking at the relative not the absolute value of different interventions.

## Methods

The mode of transmission model uses categories of those at risk of exposure: low risk are single partner couples; the casual sex group are either those having pre-marital sex or those having multiple partners; no risk are those not having sex. The mode of transmission model was populated with best estimate Malawi data for 56 of the 62 input variables required, with default estimates for Africa being used for the other six (Additional file 1). Sensitivity analysis was undertaken to assess the effect of changing the input variables for which good data are not available such as the proportion of MSM and the number of sex workers. The model was modified to take account of high infection risk during early HIV infection associated with concurrent partner transmission - a weakness of the UNAIDS model for a country like Malawi with a high sexual partner concurrency rate. This higher risk of transmission was allocated to the high risk heterosexual group (labelled casual heterosexual sex partners and their partners) in the model. It was calculated to be 2.3 times the base line risk (using data from Rakai [9]: 0.008 risk of transmission per coital act in first 5 months after infection, 0.0009 for 4 years followed by three years with risk of 0.003). The base line transmission risk from female to male was reduced such that the current circumcision rate in Uganda brought this rate to what was found in the Rakai studies. Sensitivity analysis was undertaken to assess the effect of the modification.

The model was manipulated to take into account possible HIV prevention interventions. Various scenarios were contemplated following interactive sessions with colleagues at conferences and workshops. For this study the prevalence of sexually transmitted infections (STIs) was halved in response to improved syndromic treatment services. The use of condoms with all STIs was assessed by reducing the cofactor (4 in the model) to zero. Uniform use of condoms by bar girls was assessed by increasing condom use to 100% for bar girls (from 92%) and their clients (from 64%). The circumcision rate was doubled and trebled to assess the effect on incidence in males. A 100% successful zero-grazing campaign was assessed by converting all casual and commercial sex risk to low risk adults. Doubling condom use was assessed to identify the value of this increased protection. The effect of abstinence for one year was calculated by converting 20% of the 15-24 year olds from high and low risk to no-risk.

The overall national HIV incidence per year was estimated using the Estimation and Projection Package (EPP) [12] in the latest Ministry of Health assessment [3] to be 1.6% using antenatal sero-prevalence data adjusted for the most recent demographic and health survey results carried out in Malawi in 2004 and used in the model [13]. The frequency of sexual acts was adjusted so that the overall incidence in the model was 1.6% new cases a year. This resulted in coital frequency for sexually active people to be 11 per month. This is somewhat higher than previous estimates using DHS data on time since last sexual intercourse (with one study finding of 4.4 per month) but not much more than the coital frequency in Rakai (8.9 per month) from which the transmission risk used in the model was estimated [14,15].

No participants were involved in the study and hence ethics committee approval was not required.

## Results

**Figure 1:**
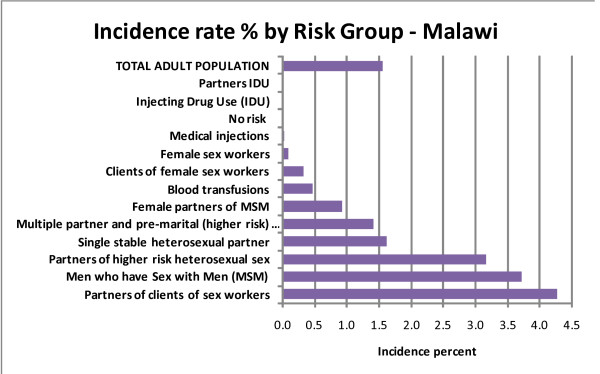
**Estimated incidence (% per year) by risk group in Malawi 2008**.

New cases in the year were estimated to be 94,454, which is an annual national incidence of 1.6% (Table 1). Non-heterosexual incident cases were very rare (Figure 1). The exposure groups with the highest incidence risk were partners of those who had high risk sex and men having sex with men (MSM). Sex workers, because so many were already infected (71%), and those who had casual sex had incidence rates below the national average. Those who had low risk sex, including single partner discordant couples, experienced the average incidence rate. Sensitivity analysis of the size of the sex worker, sex worker clients and MSM groups showed the model to be robust (Additional file 2).

**Table 1:** Incidence HIV infections in one year - Malawi using a modified model - the baseline rates

Risk category	Incident cases	Incidence %	Incidence per 100,000	Incidence rate %
Single stable heterosexual partner	34,673	36.7	1,609	1.6
Partners of higher risk heterosexual sex	25,023	26.5	3,148	3.1
Partners of clients of sex workers	16,978	18.0	4,256	4.3
Multiple partner and pre-marital (higher risk) sex	15,414	16.3	1,434	1.4
Clients of female sex workers	1,695	1.8	318	0.3
Medical injections	368	0.4	6	0.0
Blood transfusions	139	0.1	455	0.5
Men who have Sex with Men (MSM)	113	0.1	3,702	3.7
Female sex workers	38	0.0	79	0.1
Female partners of MSM	14	0.0	949	0.9
No risk	0	0.0	0	0.0
Injecting Drug Use (IDU)	0	0.0	0.0	0.0
Partners IDU	0	0.0	0	0.0
**TOTAL ADULT POPULATION**	**94,454**		**1,551**	**1.6**

**Figure 2:**
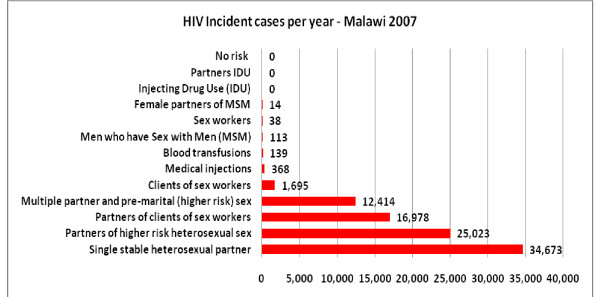
**Estimated incident numbers by risk group - Malawi 2008**.

The number of cases in each exposure group depended on its size (Figure 2). The largest group, accounting for 37% of all cases, was the low-risk heterosexual group, which includes the discordant couples. Those who had casual sex and their partners accounted for a further 43% (16% and 27% respectively) of new cases. Cases arising from commercial sex accounted for 20% of new cases, of which partners of clients dominated with 18% of all new cases. The remaining cases were accounted for by medical injections and MSM. Analysis of the effect of modifying the risk of infection in casual relationships found that the proportion of incident cases from this risk group varied from 29% if the risk of infection was similar to that of the low risk group to 52% if the risk used in the model was doubled (Additional file 2).

The results of model manipulation to assess the effect of possible HIV prevention interventions were found to produce limited effect for some - condom use with bar girls, circumcision, abstinence and reducing STIs. Major effect was found with others - condom use with discordant couples, a zero-grazing campaign, condom use with a sexually transmitted infection (STI) and in high risk sex (Table 2). The size of effect was directly related to the size of the risk group. The combination of a successful concurrent partner campaign and discordant couple campaign reduced incident cases by 99%. Even increasing condom use to 50% of occasions for discordant couples and halving the number of multiple partners would have the significant effect of reducing new infections by 36%.

**Table 2:** Effect on incident cases in Malawi following use of selected prevention interventions

Intervention to reduce new infections from 94,454		number of new infections per year averted	percent reduction in new infections
1	100% condoms for discordant single partner couples	34,673	37%
2	100% multiple concurrent (casual and commercial) partnerships become monogamous	16,668	18%
3	100% Condom use when STI	13,570	14%
4	Double condom use in high risk sex from 43.1% to 86.2%	11,501	12%
5	20% sexually active 15-24 year olds abstain	7,932	8%
6	Treble circumcision from 21% to 63%	7,512	8%
7	Improve STI services to reduce STIs by half	6,785	7%
8	Double circumcision from 21% to 42%	3,765	4%
9	All bar girls use condoms all the time	1,731	2%
**Multiple interventions to reduce new infections from 94,454**			
10	100% condoms for discordant couples and 100% monogamy (1 & 2 combined)	93,858	99%
11	50% condoms for discordant couples and 50% multiple partnerships become monogamous	33,986	36%

## Discussion

The mode of transmission model is easy to use and adapt to the local situation. Sufficient local data were found to populate 90% of necessary model variables. The use of such data and a simple model gave national planners confidence to base a new HIV prevention strategy on the results. The results were sufficiently different from preconceived notions of which population groups had the most new infections to make a big shift in strategic emphasis. It appears that while originally developed for low level epidemics the mode of transmission model can be used in generalised epidemics if sufficient local data are available as found in this study. The UNAID Estimation and Projection Package (EPP) provides estimates of overall levels of HIV transmission. The mode of transmission model provides estimates of the relative contribution of the different risk groups.

The predominant data set was from the most recent DHS. This has significant problems of interpretation in relation to recall of sexual activity, on which the model relies. In this DHS which had an HIV test refusal rate of 22%, the recall of sexual activity about coital frequency and multiple partners are particular causes of uncertainty. Fortunately Malawi has some detailed sexual activity data collected by the Malawi Diffusion and Ideation Project over a number of years [16-19]. This information helped to modify DHS data where appropriate. Despite the limitations of the data the model does produce results which are consistent with other estimates of HIV incidence.

However the main limitation of the study is the sensitivity of the choice of risk of infection in the different risk groups. Until evidence is available to be confident about the proportion of concurrent partners and new infections that make up the casual risk group, any analysis relies on the assumptions used in the model. The model can be re-run with revised assumptions as this evidence becomes available. An advantage of the model over more sophisticated ones is that public health practitioners can test quite easily the sensitivity of the model to changes in input variables about which they are unsure. Indeed the model is simple enough to be used in planning meetings where participants can suggest alternative estimates of input variables and alternative mixes of possible interventions.

The choice of intervention depends on the relative importance of the mode of transmission with which it interferes, its effectiveness and its cost. The cost-effectiveness of HIV/AIDS interventions is known [20]. While cost might seem an impediment, despite its poverty Malawi is willing to spend considerable sums of money on one HIV related intervention - namely the use of antiretroviral therapy (ART). There are many cost effective HIV/AIDS interventions which save more lives for the same cost. There should be no problem obtaining donor funds for any of these better value interventions if felt necessary for a successful HIV prevention strategy.

But which HIV prevention interventions are effective in the Malawian setting? The choice of appropriate interventions requires knowledge and experience of ways which are likely to change sexual behaviour in the Malawi population. Clearly interventions used to date have been inadequate, insufficient or inappropriate. A revitalised strategy will need to include more of those existing strategies that have worked and new ones such as a "zero grazing campaign" and couple HCT which tackle the two modes of transmission now found to be so important in Malawi - **concurrency and discordancy**.

## Conclusion

In conclusion the mode of transmission model offers a practical means of identifying in countries such as Malawi with a generalised HIV epidemic those groups within the community for which HIV prevention programmes can be targeted.

## Competing interests

The authors declare that they have no competing interests.

## Authors' contributions

KM undertook the compilation of data sources for input variables and populated the model. CB modified the model, undertook the analysis of possible interventions and wrote the first draft of the manuscript for publication. Both authors read and approved the final manuscript.

## Pre-publication history

The pre-publication history for this paper can be accessed here:

http://www.biomedcentral.com/1472-6963/10/243/prepub

## Supplementary Material

Additional file 1 [21-30]**Data sources used to populate the Mode of Transmission model - Malawi 2007**. Data sources referenced which were used to populate the Mode of Transmission model demonstrating the availability of most data items from local data sources and the limited number variables for which regional estimates were used. Click here for file

Additional file 2 **Sensitivity analysis of Mode of Transmission results**. Sensitivity analysis assessing the effect on HIV incidence using Mode of Transmission model by varying estimates of risk of transmission variable and size of selected risk groups - Malawi 2008. Click here for file
