# Supplementary material for: Selecting HIV infection prevention interventions in the mature HIV epidemic in Malawi using the mode of transmission model
Source: BMC Health Serv Res. 2010 Aug 19;10:243. doi: 10.1186/1472-6963-10-243 (PMC2936376; doi:10.1186/1472-6963-10-243)
Supplement: Additional file 1 — [21-30]Data sources used to populate the Mode of Transmission model - Malawi 2007. Data sources referenced which were used to populate the Mode of Transmission model demonstrating the availability of most data items from local data sources and the limited number variables for which regional estimates were used. [file 1472-6963-10-243-S1.DOC]

**Data sources used to populate the Mode of Transmission model - Malawi 2008**

| **Risk group** | **Estimate** | **Year of estimate** | **Data sources / comments** | **Reference** |
| --- | --- | --- | --- | --- |
| **Total number of adults (15-49 years)** | 6,090,217 | 2007 | National Statistics Office Census 1998 projected to 2007 |  |
| Number of Males | 2,959,622 | 2007 |  |  |
| Number of Females | 3,130,595 | 2007 |  |  |
| Estimated Adult HIV prevalence | 12.7 | 2007 | Based on DHS 2004 HIV prevalence using Spectrum model | [3] |
| Number / percent Injecting Drug Use in the adult population: | 0% |  | None known in Malawi |  |
| **Sex workers*** |  |  |  |  |
| Number / percent of female sex workers in the population FSW) | 1.58% | 2006 | 1.58% attended CSW clinics in Thyolo. Default for Sub-Saharan Africa - 0.4-4.3%; note: Zambia 2.4% in Ndola, 2.7% border areas with Malawi. | [21; 22] |
| Prevalence of HIV in this population | 70.7% | 2006 | Malawi Biological and Behavioural Surveillance Survey (BBSS) 2006 | [23] |
| Prevalence of any STI in this population | 25% | 1999 | Thyolo study | [21] |
| Average number of sexual partners per year | 44.9 | 2006 | BBSS 2006 mean number of sexual partners | [23] |
| Average number of sexual acts per partner per year | 3.2 | 1999 | BBSS Zambia Female Sex Workers 2000 - 2.8 clients per week. With 44.9 partners a year this means frequency per partner is 3.2 | [24] |
| Percentage of times a condom is used during sex | 91.8% | 2006 | BBSS 2006 - % used condom last time they engaged in commercial sex in the last 12 months | [23] |
| **Clients of sex workers*** |  |  |  |  |
| Percent of sex worker clients in the population | 17.5% | 2004 | Assume 3.5 times higher than DHS 2004: 5% men aged 15 - 49 had sex with CSW as serious under reporting. Malawi Diffusion and Ideational Change Project (MDIC) suggests much higher prevalence - approx 40%. | [13; 25] |
| Prevalence of HIV in this population | 17% | 2004 | DHS 2004: 17% among men with 2 or more partners; 11.3% among those that paid for sex past 12 months; 14.2% among truck drivers and 19.9% in male estate workers in BBSS 2006 | [13; 23] |
| Prevalence of any STI in this population | 7.3% | 2004 | DHS 2004: 5.6% self reported any STI symptom among all men; 8 % among truck drivers BBSS 2004. Community survey 6.1% | [13; 23; 26] |
| Average number of sex worker partners per year | 3.8 | 2000 | MDIC surveys suggest average sex with FSW monthly once a month. Requires 3.8 if frequency per FSW is 3.2 | [25] |
| Average number of sexual acts per partner per year | 3.2 | 1999 | As FSW - see above |  |
| Percentage of times a condom is used during sex with a sex worker | 63.6% | 2006 | DDSS Estate workers with FSW | [23] |
| **Partners of Clients*** |  |  |  |  |
| Percent of partners of sex worker clients in the population | 13.1% | 2004 | DHS2004 - of those having paid for sex in last year 25% were neither married or had a partner - so 75% of 17.5% = 13.1% | [13] |
| Prevalence of HIV in this population | 13.2% | 2004 | DHS2004 | [13] |
| Average number of sexual partners per year | 1 | 2004 | DHS2004 - Table 11.8 | [13] |
| Average number of sexual acts per partner per year | 132 |  | As low-risk partner frequency – see below |  |
| Percentage of times a condom is used during sex | 10 | 2004 | DHS2004 | [13] |
| **Men who have sex with men (MSM)*** |  |  |  |  |
| Percent of MSM in the population | 0.1% |  | Default values used |  |
| Prevalence of HIV in this population | 20% | 2008 | Snowball survey in Blantyre and Lilongwe 2008 | [27] |
| Prevalence of any STI in this population | 15% |  | Default values used |  |
| Average number of sexual partners per year | 4.2 | 2008 | Snowball survey in Blantyre and Lilongwe 2008 - 6 month recall | [27] |
| Average number of sexual acts per partner per year | 10 |  | Default values used |  |
| Percentage of times a condom is used during sex | 60% | 2008 | Snowball survey in Blantyre and Lilongwe 2008 - 6 month recall | [27] |
| **Female partners of MSM*** |  |  |  |  |
| Percent of female partners of MSM in the population | 0.047% | 2008 | Snowball survey in Blantyre and Lilongwe 2008 - 47% of MSM are married or have a regular female partner | [27] |
| Prevalence of HIV in this population | 13.2% | 2004 | DHS2004 | [13] |
| Average number of sexual partners per year | 1.5 | 2008 | Snowball survey in Blantyre and Lilongwe 2008 - 6 month recall | [27] |
| Average number of sexual acts per partner per year | 66 |  | Half the low-risk partnership frequency – see below |  |
| Percentage of times a condom is used during sex | 63.5% | 2008 | Snowball survey in Blantyre and Lilongwe 2008 - 6 month recall | [27] |
| **Casual heterosexual sex*** |  |  |  |  |
| Percent of people in the general population who engage in casual sex (i.e. non-regular, non-cohabiting sex, or having multiple sex partners): |  |  |  |  |
| Male | 26.9% | 2004 | DHS 2004 | [13] |
| Female | 8.3% | 2004 | DHS 2004 | [13] |
| Prevalence of HIV in this population | 12.4% | 2004 | DHS 2004 | [13] |
| Prevalence of any STI in this population | 9.5% | 2004 | 1.7 times (9%) self reported STI by high risk men as compared to (6%) sexually active men in the last 12 months in DHS2004 | [13] |
| Average number of sexual partners per year | 1.4 | 2004 | DHS 2004 | [13] |
| Average number of sexual acts per partner per year | 66 | 2005 | Half the low-risk partnership frequency – assumed by MDIC project from their surveys | [25] |
| Percentage of times a condom is used during sex | 43.1% | 2004 | DHS 2004; 47.1% in men and 30.1% in women | [13] |
| **Partners CHS*** |  |  |  |  |
| Percent of people in the general population whose partners engage in casual sex: |  |  |  |  |
| Male | 7.5% | 2004 | DHS 2004 - 0.8% of married women had higher risk sex | [13] |
| Female | 18.6% | 2004 | DHS 2004 - 8.3% of married men had higher risk sex | [13] |
| Prevalence of HIV in this population | 12.40% | 2004 | DHS 2004 people who had higher risk sex | [13] |
| Average number of sexual partners per year | 1 |  |  |  |
| Average number of sexual acts per partner per year | 132 |  | As low-risk partners – see below |  |
| Percentage of times a condom is used during sex | 18.7% | 2004 | DHS 2004 - married men with higher risk sex last intercourse with wife using condom | [13] |
| **Low-risk heterosexual sex*** |  |  |  |  |
| Percent of people in stable relationships (i.e., married or in a relationship with one stable partner) : | M - 29.8% F – 41.0% |  | Lowest category of risk for remaining population |  |
| Prevalence of HIV in this population | 13% | 2004 | DHS 2004: 11.6 in non-polygamous union for females and 14.5% for men; total 13% | [13] |
| Prevalence of any STI in this population | 5.6% | 2004 | Community based survey – 6.1% males (gonorrhoea) and 5% females (Chlamydia) | [26] |
| Average number of sexual partners per year | 1 |  |  |  |
| Average number of sexual acts per partner per year | 132 |  | This is 11 coital acts per month. Rakai study from which the transmission risks were calculated had coital frequency of 8.9 per month. DHS 2004 finds low risk female coital frequency of 104 per year and men 100 but recall is suspect. Duration of post-natal abstinence is falling from reports on cultural practices. | [13; 28; 15] |
| Percentage of times a condom is used during sex | 3.8% | 2004 | DHS 2004 - males 4.5% and females 3.1% | [13] |
| **No risk** |  |  |  |  |
| Percent of people who are at NO risk of HIV infection |  |  |  |  |
| male | 18.1% | 2004 | DHS 2004 - never intercourse or abstinence for over 12 months | [13] |
| female | 17.4% | 2004 | DHS 2004 - never intercourse or abstinence for over 12 months | [13] |
| **Medical injections** |  |  |  |  |
| Prevalence of HIV in this population | 12.7% | 2007 | Malawi estimate 2007 - national prevalence | [3] |
| Average number of medical injections received per person per year | 0.8 | 2004 | DHS 2004 - 0.8 per person | [13] |
| Percentage of times sterile needles are used for medical injections | 83% |  | Default estimate for S & E Africa | [29] |
| **Blood transfusions** |  |  |  |  |
| Number of people receiving blood transfusions per year | 0.5% |  | Default value used - E & S Africa: 5/1000 per year |  |
| Prevalence of HIV in this population | 12.7% | 2007 | Malawi estimate 2007 - national prevalence | [3] |
| Average number of blood transfusions per year (i.e. for those people who received a blood transfusion) |  | 2007 | Malawi Blood Transfusion Service - average of 1 |  |
| Percentage of bloods that are effectively screened for HIV | 96% |  | Default value used - Global WHO database for S&E Africa: 96% Published studies: 72-93% |  |
| **Transmission risk** |  |  |  |  |
| Long term relations - female to male | 0.0007 | 1999 |  | [15] |
| Long term relations - male to female | 0.0011 | 1999 |  | [15] |
| Short term relations - female to male | 0.0016 | 1999 | Base line transmission risk increased by 2.3 3 to take account for high viral load more likely in new relationships | [9] |
| Short term relations - male to female | 0.0026 | 1999 |  | [9] |
| STI cofactor | 4 | 2001 |  | [15] |
| Percent of adult males that are circumcised | 20.7% | 2004 | DHS 2004, giving a 60% protection to males | [13; 30] |
| Notes: * A person is only counted once, and is placed in the category of highest HIV risk (excluding medical injections and blood transfusions)  # All DHS 2004 HIV prevalence estimates adjusted by 1.15 to account for under-representative sample and high refusal rate of 22%, bringing model total 15-49 population to 12.7% - the nationally adopted rate. | | | | |
