# Supplementary material for: Selecting HIV infection prevention interventions in the mature HIV epidemic in Malawi using the mode of transmission model
Source: BMC Health Serv Res. 2010 Aug 19;10:243. doi: 10.1186/1472-6963-10-243 (PMC2936376; doi:10.1186/1472-6963-10-243)
Supplement: Additional file 2 — Sensitivity analysis of Mode of Transmission results. Sensitivity analysis assessing the effect on HIV incidence using Mode of Transmission model by varying estimates of risk of transmission variable and size of selected risk groups - Malawi 2008. [file 1472-6963-10-243-S2.DOC]

Sensitivity analysis assessing the effect on HIV incidence using Mode of Transmission model by varying estimates of risk of transmission variable and size of selected risk groups – Malawi 2008

| **Multiplication factor for** | **Incidence of new infections in** | **Percent change from baseline of incidence in** | **Proportion of overall incidence held by risk group** |
| --- | --- | --- | --- |
|
| **risk of transmission in casual partnerships** | **Casual partnerships** | | |
| 0.4 | 21750 | 54% | 31.9% |
| 1.0 | 40437 | 100% | 42.8% |
| 1.5 | 56778 | 140% | 48.6% |
| 2.0 | 71262 | 176% | 52.2% |
| **size of sex worker population (1.58% in model)** | **Sex workers** | | |
| 0.5 | 19 | 50% | 0.02% |
| 1.0 | 38 | 100% | 0.04% |
| 1.5 | 57 | 150% | 0.06% |
| 2.0 | 76 | 200% | 0.08% |
| **size of sex worker client group (17.5% in model)** | **Clients of sex workers** | | |
| 0.5 | 847 | 50% | 0.90% |
| 1.0 | 1695 | 100% | 1.80% |
| 1.5 | 2542 | 150% | 2.80% |
| 2.0 | 3389 | 200% | 3.80% |
| **size of MSM group (0.1% in model)** | **MSM** | | |
| 0.5 | 56 | 50% | 0.06% |
| 1.0 | 113 | 100% | 0.12% |
| 1.5 | 169 | 150% | 0.18% |
| 2.0 | 225 | 199% | 0.24% |
| 5.0 | 564 | 499% | 0.60% |
